# Carbonic Anhydrase from *Porphyromonas Gingivalis* as a Drug Target

**DOI:** 10.3390/pathogens6030030

**Published:** 2017-07-15

**Authors:** Claudiu T. Supuran, Clemente Capasso

**Affiliations:** 1 Dipartimento Neurofarba, Sezione di Scienze Farmaceutiche, and Laboratorio di Chimica Bioinorganica, Polo Scientifico, Università degli Studi di Firenze, Via U. Schiff 6, Sesto Fiorentino, 50019 Florence, Italy; 2 Istituto di Bioscienze e Biorisorse, CNR, via Pietro Castellino 111, 80131 Napoli, Italy

**Keywords:** periodontitis, *Porphyromonas gingivalis*, carbonic anhydrases, sulfonamides, anions, kinetic constants, antiinfectives, antibacterials

## Abstract

Periodontitis originates from a microbial synergy causing the development of a mouth microbial imbalance (dysbiosis), consisting of a microbial community composed of anaerobic bacteria. Most studies concerning the treatment of periodontitis have primarily take into account the Gram-negative bacterium *Porphyromonas gingivalis*, because it is a prominent component of the oral microbiome and a successful colonizer of the oral epithelium. Here, we focus our attention on the study of the carbonic anhydrases (CAs, EC 4.2.1.1) encoded in the genome of this pathogen as a possible drug target. Carbonic anhydrases are a superfamily of metalloenzymes, which catalyze the simple but physiologically crucial reaction of carbon dioxide hydration to bicarbonate and protons. Bacterial CAs have attracted significant attention for affecting the survival, invasion, and pathogenicity of many microorganisms. The *P. gingivalis* genome encodes for two CAs belonging to β-CA (PgiCAβ) and γ-CA (PgiCAγ) families. These two enzymes were cloned, heterologously expressed in *Escherichia coli*, and purified to homogeneity. Moreover, they were subject to extensive inhibition studies using the classical CA inhibitors (sulfonamides and anions) with the aim of identifying selective inhibitors of PgiCAβ and PgiCAγ to be used as pharmacological tools for *P. gingivalis* eradication.

## 1. Introduction

The most aggressive disease involved in the degradation of the periodontium, the structure supporting the teeth, is known as periodontitis [1]. For humans, the great risks associated with this disease are not only the loss of teeth but also the development of atherosclerosis, rheumatoid arthritis, aspiration pneumonia, and cancer [1]. The microbial community of the human mouth is colonized by more than 700 different species of bacteria [2]. Mouth microbiota strongly influences the development and maintenance of immune homeostasis, acts as a barrier against pathogen invasion, and provides the host with nutritional contributions [1,2]. The healthy mouth microbiota is composed mostly of facultative bacterial genera belonging to the Actinomyces and Streptococci [1,2]. It has been reported that periodontitis results not from individual pathogens but from a microbial synergy causing the development of a mouth microbial imbalance (dysbiosis), which consists of a microbial community composed of anaerobic genera belonging to the phyla Firmicutes, Proteobacteria, Spirochaetes, Bacteroidetes, and Synergistetes [1,2]. The dysbiosis generates periodontopathogens, destroys the mouth mutualistic relationships, and influences host physiology, compromising the periodontal tissue homeostasis and the human health status. Among the periodontopathogens, the species *Porphyromonas gingivalis*, *Treponema denticola*, and *Tannerella forsythia* were considered the main candidates for the clinical destruction of the gingiva, periodontal ligament, and alveolar bone, which are elements of the teeth-supporting tissues [2]. A serious problem associated with periodontitis is that the polymicrobial community is usually resistant to antimicrobial agents and host-defense mechanisms. In this context, we investigated the possibility of finding new anti-infectives by studying the inhibition profiles of carbonic anhydrases (CAs, EC 4.2.1.1), a superfamily of metalloenzymes which catalyze the simple but physiologically crucial reaction of carbon dioxide hydration to bicarbonate and protons: CO_2_ + H_2_O ⇄ HCO_3_^−^ + H^+^ [3,4,5,6,7,8,9,10,11]. Moreover, most studies concerning the treatment of periodontitis have primarily take into account the Gram-negative bacterium *Porphyromonas gingivalis* because it is a prominent component of the oral microbiome and a successful colonizer of the oral epithelium [2]. Thus, since *Porphyromonas gingivalis* is the main etiological agent present in severe forms of periodontitis, we focused our attention on the study of the carbonic anhydrases encoded by the genome of this pathogen as a possible drug target [10].

### 1.1. Virulence of Porphyromonas Gingivalis

*Porphyromonas gingivalis* is a Gram-negative anaerobic, rod-shaped, immobile, and asaccharolytic bacterium. It is able to infect gingival epithelial cells, periodontal ligament fibroblasts, and alveolar osteoblasts [2,12]. The infection caused by this bacterium influences the modulation of host immune inflammatory responses altering the periodontal microbiota [1,12]. The virulence of the periodontopathogens is governed by different factors: (*i*) the fimbriae and hemagglutinins, which are colonization factors determining for the tissue invasion; (*ii*) the gingipains, which are cysteine proteinases causing edema, neutrophil infiltration, and the degradation of fibrinogen; (*iii*) lipopolysaccharides, which are associated with increased matrix metalloproteinases and initiate an inflammatory cascade involving reactive oxygen species, proinflammatory cytokines, and matrix metalloproteinases (MMP); and (*iv*) outer membrane vesicles containing lipopolysaccharides and gingipains [1,12]. Moreover, the pro-inflammatory strategies adopted by *Porphyromonas gingivalis* are connected to chronic inflammation in extra-oral sites. Periodontitis, in fact, may be associated with atherosclerotic cardiovascular disease, an increased risk of adverse pregnancy outcomes, rheumatoid arthritis, and respiratory diseases [1,12]. The most important aspect is that *Porphyromonas gingivalis* and the polymicrobial community represent a significant health risk due to their resistance to host defense mechanisms as well as their resistance to conventional antimicrobials [1,12]. In the search for novel molecular targets that are capable of being inhibited by new anti-infectives and can therefore be targeted to combat periodontitis, we investigated the genome of *Porphyromonas gingivalis* for the presence of genes encoding for CAs [13,14,15]. The genome of *P. gingivalis* encodes for a β- and a γ-CA. Thus, our groups cloned, expressed, and purified the β-CA (named PgiCAβ) and γ-CA (named PgiCAγ) identified in the genome of this pathogenic bacterium [10,16,17,18,19,20,21,22,23,24,25]. These two proteins were biochemically characterized and extensively investigated for their inhibition profiles towards sulfonamides and anions.

### 1.2. Carbonic Anhydrases

CAs are ubiquitous metalloenzymes with the catalytically active form represented by the metal hydroxide derivative [6,10,26,27,28]. CAs are grouped in seven genetically distinct families, named α-, β-, γ-, δ-, ζ-, η- and ɵ-CAs, with a similar structure and hydratase activity, but low sequence similarity [4,6,10,14,15,26,27,28,29,30,31,32,33,34,35]. The α-, β-, δ-, η- and, perhaps θ-CAs use as a catalytic metal the Zn(II) ion. γ-CAs are Fe(II) enzymes, but they are active also with bound Zn(II) or Co(II) ions [36,37,38,39,40,41,42,43], while ζ-CAs bind Cd(II) or Zn(II) within the active site and are defined as cambialistic enzymes [44,45,46]. The metal ion from the CA active site is coordinated by three His residues in the α-, γ-, δ- and probably the θ-classes; it is coordinated by one His and two Cys residues in β- and ζ-CAs; or by two His and one Gln residues in the η-class, with the fourth ligand being a water molecule/hydroxide ion acting as a nucleophile in the catalytic cycle of the enzyme [10,13,15,47,48,49,50]. The rate-determining step of the entire catalytic process is the formation of the metal hydroxide species of the enzyme by the transfer of a proton from the metal-coordinated water molecule to the surrounding solvent [10,13,14,15,26]. All CAs identified in animal systems belong to the α-class. CAs identified in plants and algae belong to the α-, β-, γ-, δ- and θ-classes; fungi encode for α- and β-CAs; protozoa encode for α-, β- or η-CAs. Bacteria encode for enzymes belonging to the α-, β- and γ-CA classes [10,13,14,15,29,51,52]. CAs are involved in many crucial physiologic and pathologic processes connected to pH regulation, secretion of electrolytes, biosynthetic processes, photosynthesis, tumorigenesis, etc. In bacteria, the existence of genes encoding for CAs from at least one of the three classes (α, β, and γ) suggests that these enzymes play an important role in the bacterial physiology. In fact, it has been demonstrated that bacterial CAs are involved in the transport and supply of CO_2_ or HCO_3_^-^ and pH regulation. Recently, in fact, bacterial CAs have attracted significant attention for influencing the growth of microorganisms [6,10,26,27,28] because they affect the survival, invasion, and pathogenicity of the microorganism. [53,54,55,56,57,58,59,60,61,62]. For example, the α- and β-CAs identified in the genome of *Helicobacter pylori* play a crucial role in the acid acclimatization of the pathogen within the stomach [53,54]; also, the α-, β-and γ-CAs of *Vibrio cholerae* are involved in the production of sodium bicarbonate, which induces cholera toxin expression [63,64]. Fascinatingly, the addition of CA inhibitors caused an eradication of *H. pylori* from the stomach and, for *Vibrio cholerae*, a significant reduction in virulence gene expression [53,54,63,64]. In fact, the *in vivo* inhibition of bacterial CAs influences the pathogenicity and/or the growth of the microorganism [13,53,56,60,65,66,67]. Moreover, CAs are involved in the cyanate degradation of *Escherichia coli*.

### 1.3. Carbonic Anhydrase Inhibitors

Different types of CA inhibitors (CAIs) exist [44,68]. They can be grouped according to whether they bind to a catalytic metal ion or metal coordinated-water molecule, and according to the occlusion of their active site. Following this criterion, at least four groups of CAIs have been identified: (1) the metal ion binders (anion, sulfonamides and their bioisosteres, dithiocarbamates, xanthates, etc.); (2) compounds which anchor to the zinc-coordinated water molecule/hydroxide ion (phenols, polyamines, thioxocoumarins, sulfocumarins); (3) compounds occluding the active site entrance, such as coumarins and their isosteres; and (4) compounds binding out of the active site. Sulfonamides and anions are the best-investigated inhibitors of bacterial carbonic anhydrases.

#### 1.3.1. Anions

Anions, such as inorganic metal-complexing anions or more complicated species such as carboxylates, are also known to bind to CAs [44,68]. These anions may bind either the tetrahedral geometry of the metal ion or as trigonal–bipyramidal adducts. Anion inhibitors are important both for understanding the inhibition/catalytic mechanisms of these enzymes fundamental for many physiologic processes, and for designing novel types of inhibitors which may have clinical applications for the management of a variety of disorders in which CAs are involved [44,68].

#### 1.3.2. Sulfonamides

In 1935, Domagk discovered sulfonamides [69], which were the first antimicrobial drugs. The first sulfonamide showing effective antibacterial activity was Prontosil, a sulfanilamide prodrug isosteric/isostructural with p-aminobenzoic acid (PABA) [70]. In the following years, a range of analogs constituting the so-called sulfa drug class of anti-bacterials entered into clinical use, and many of these compounds are still widely used. Sulfonamides, such as the clinically used derivatives acetazolamide, methazolamide, ethoxzolamide, dichlorophenamide, dorzolamide, and brinzolamide, bind in a tetrahedral geometry to the Zn(II) ion in the deprotonated state, with the nitrogen atom of the sulfonamide moiety coordinated to Zn(II) and an extended network of hydrogen bonds, involving amino acid residues of the enzyme, also participating in the anchoring of the inhibitor molecule to the metal ion [44,47,68,71]. The aromatic/heterocyclic part of the inhibitor interacts with the hydrophilic and hydrophobic residues of the catalytic cavity.

## 2. Sequence Analysis

The full nucleotide sequence of the gene encoding for PgiCAβ showed an open reading frame encoding for a polypeptide chain of 242 amino acid residues, which contained all the typical features of a β-CAs: the three residues that are involved in the catalytic mechanism of the enzyme (two cysteines and one histidine), and the catalytic dyad formed by one aspartate and one arginine residue involved in the activation of the zinc-coordinated water molecule for the nucleophilic attack [10,25]. Intriguingly, we noted that the primary structure of β-CAs identified in the genome of the Gram-negative *P. gingivalis* present a pre-sequence of 18 or more amino acid residues at the N-terminal part, which was characterized as a signal peptide [6,15]. Thus, it was hypothesized that *P. gingivalis* β-CAs might have a periplasmic localization and a role similar to that described previously for α-CAs. The open reading frame of the *P. gingivalis* γ-CA gene encoded for a 192-amino acid polypeptide chain (PgiCAγ), displaying an identity from 33 to 30% when compared with the prototypical γ-CAs represented by the acronyms CAM, CAMH, and CcmM, and extensively studied from Ferry’s group [23,72,73,74]. CAM and CAMH were identified and isolated from the archaeon *Methanosarcina thermophila*, while CcmM was isolated from the thermophilic bacteria *Thermosynechococcus elongates*. A comparative sequence analysis of these γ-CAs showed that the three histidine residues involved in the Zn(II) coordination are conserved in PgiCAγ. Interestingly, the proton shuttle residue (Glu 84), a distinctive hallmark of the CAMH protein, was missed in PgiCAγ, suggesting that this protein probably does not belong to the CAMH type. The results of Ferry’s group suggest that the majority of the γ-class CAs belong to the CAMH type [10,16,17,18,19,20,21,22,23,24,25].

## 3. Biochemical Characterization

Isopropyl β-D-1-thiogalactopyranoside (IPTG) induction of *Escherichia coli* BL21 (DE3) cells transformed with the plasmid pET15-b/PgiCAβ or pET15-b/PgiCAγ resulted in the production of the recombinant PgiCAβ or PgiCAγ [10,16,17,18,19,20,21,22,23,24,25]. The recombinant *P. gingivalis* CAs were isolated and purified to homogeneity from *E. coli* (DE3) cell extract. Most of the CA activity was recovered in the soluble fraction of cell extract after sonication and centrifugation. Using the affinity column (His-select HF Nickel Affinity gel), the recombinant protein (PgiCAβ or PgiCAγ) was purified to an apparent homogeneity. PgiCAγ showed a subunit molecular mass of 21 kDa estimated by SDS-PAGE, while SDS-PAGE of PgiCAβ showed two main bands of about 25 kDa (monomeric form) and 50 kDa (dimeric form) under reducing conditions. Recombinant PgiCAβ and PgiCAγ were subject to HPLC (High-Performance Liquid Chromatography) size exclusion chromatography under native conditions to determine the oligomeric state of the protein [16,23,25]. The HPLC gel-permeation chromatography of the PgiCAβ showed a peak of activity at a molecular weight of 50 kDa. This is in agreement with the biological functional unit of β-CAs, which appears to be a dimer, a tetramer, or an octamer. HPLC gel-filtration chromatography of PgiCAγ gave an estimated molecular mass of 65 kDa [16,23,25]. Given a calculated subunit molecular mass of 21 kDa, these results suggest that the recombinant enzyme self-associates in a homotrimer. This is in agreement with previous data obtained on the γ-class CAM (γ-CA from *Methanosarcina thermophila*), which confirmed that the CAM exhibits a homotrimer structure with an approximate molecular mass of 70 kDa (data reported by Ferry’s group) [72,73,75]. The effect of the temperature on the stability of PgiCAγ has also been investigated, by incubating the enzyme at temperatures ranging from 25 to 90 °C for an incubation period time up to 2 h. It was found that the enzyme was stable up to 80 °C when the incubation time did not exceed 30 min.

## 4. Catalytic Properties

The kinetic parameters for the physiologic reaction, i.e. CO_2_ hydration to bicarbonate and protons, were determined for the purified recombinant PgiCAβ and PgiCAγ using the stopped-flow techniques (Table 1) [24,25]. PgiCAβ showed significant catalytic activity with a k_cat_ of 2.8 × 10^5^ s^−1^ and a k_cat_/K_m_ of 1.5 × 10^7^ M^−1^ s^−1^, while PgiCAγ had a k_cat_ of 4.1 × 10^5^ s^−1^ and a k_cat_/K_m_ of 5.4 × 10^7^ M^−1^ s^−1^. Table 1 shows a comparison of the kinetic parameters of PgiCAβ and PgiCAγ with those of other CAs belonging to different families and from different organisms. CAs belonging to the α-class resulted in the fastest CAs known to date, i.e., SazCA from the extremophilic bacterium *Sulfurihydrogenibium azorense* showed a k_cat_ of 4.4 × 10^6^ s^−1^; whereas the β- and γ-CAs showed kinetic constants ranging from 10^4^ to 10^5^ s^−1^. Interesting, PgiCAβ was about 2.3 times faster than the β-CA isolated from *Flaveria bidentis* (FbiCA1) and 1.5 times slower when compared with PgiCAγ [24,25].

## 5. Sulfonamide Inhibition Studies

**Figure 1:**
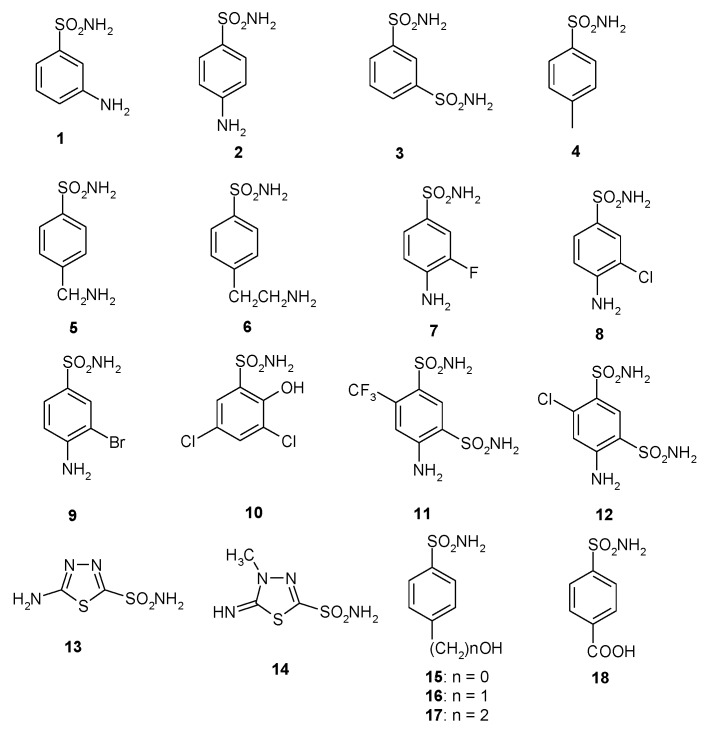

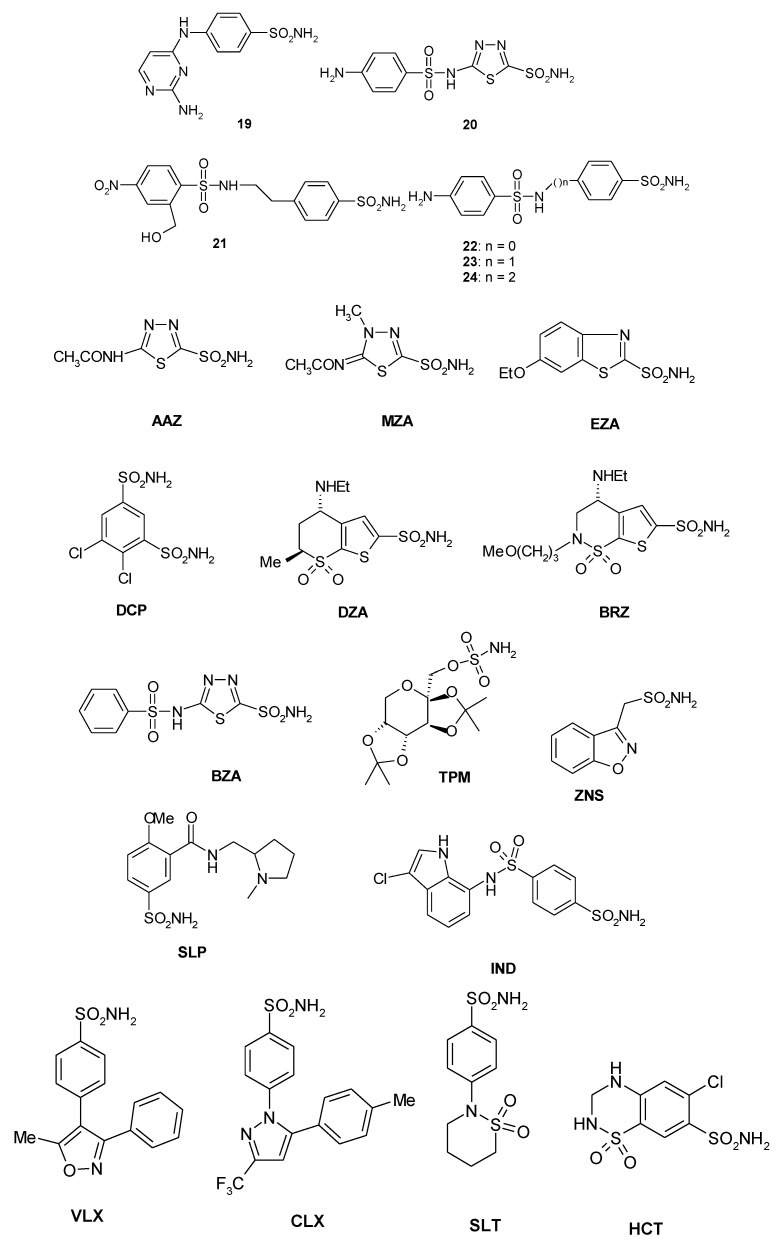
Structure of the sulfonamides/sulfamates investigated.

A library of 40 compounds, 39 sulfonamides and one sulfamate, were used in this study (Figure 1) [4,9,11,22,27,33,67,76,77,78,79]. Derivatives **1–24** and **AAZ-HCT** are either simple aromatic/heterocyclic sulfonamides widely used as building blocks for obtaining new families of such pharmacological agents, or they are clinically used agents, among which acetazolamide (**AAZ**), methazolamide (**MZA**), ethoxzolamide (**EZA**), and dichlorophenamide (**DCP**) are the classical, systemically acting antiglaucoma CAIs. Dorzolamide (**DZA**) and brinzolamide (**BRZ**) are topically acting antiglaucoma agents; benzolamide (**BZA**) is an orphan drug belonging to this class of pharmacological agents; topiramate (**TPM**), zonisamide (**ZNS**), and sulthiame (**SLT**) are widely used antiepileptic drugs. Sulpiride (**SLP**) and indisulam (**IND**) were also shown by our group to belong to this class of pharmacological agents, together with the COX2 selective inhibitors celecoxib (**CLX**) and valdecoxib (**VLX**). Saccharin (**SAC**) and the diuretic hydrochlorothiazide (**HCT**) are also known to act as CAIs.

**Figure 2:**
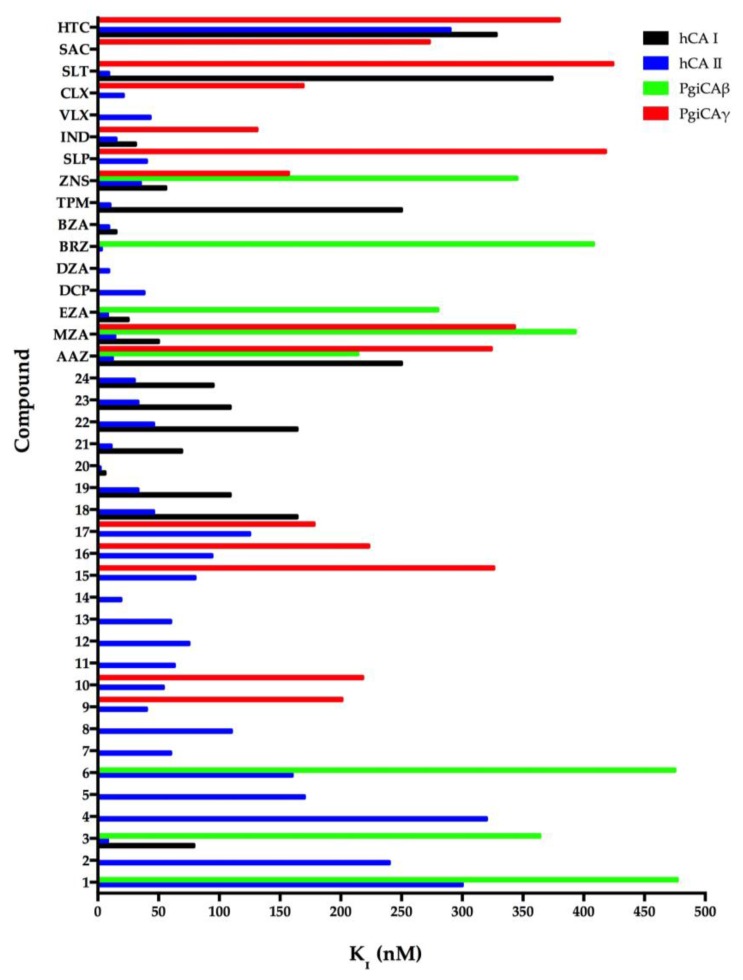
Inhibition of human isoforms hCA I and hCA II, and of the two *Porphyromonas gingivalis* CAs (PgiCAβ and PgiCAγ) with sulfonamides **1–24** and the clinically used drugs **AAZ-HTC** for the CO_2_ hydration reaction. The figure was generated using the program Prism and excludes all the inhibitors with K_I_ > 500 nM. The absence of the bar means that the inhibitor exhibits K_I_ > 500 nM. The errors were in the range of 5–10% of the shown data from three different assays.

In general, the two recombinant PgiCAβ and PgiCAγ were antithetic to the sulfonamides inhibitors because the effective inhibitors of PgiCAβ were ineffective towards PgiCAγ, and *vice versa* [17,20]. However, the behavior of the pharmacological inhibitor **ZNS** with a K_I_ of 345 nM against PgiCAβ and 157 nM against PgiCAγ was intriguing. In fact, compounds **1–7**, **MZA**, and **BRZ** had inhibition constants ranging between 345 and 818 nM towards PgiCAβ, but were rather ineffective as PgiCAγ inhibitors. Moreover, **AAZ** and **EZA** were the most effective **PgiCAβ** inhibitors, while compounds **9**, **10**, **16**, **ZNS**, and **IND** were very effective against PgiCAγ. Figure 2 was generated excluding all the inhibitors with Ki > 500 nM. As shown in Figure 2, it is readily apparent that PgiCAγ (red bars in the graph) showed more efficient inhibitors with respect to PgiCAβ (green bars in the graph). We also considered the inhibition profile of hCA I and hCA II. These two human CAs were inhibited efficiently by large numbers of sulfonamides (Figure 2). In the figure, the shorter the colored bar, the more efficient the inhibitor. It is evident that to eradicate *P. gingivalis* infection, it is necessary to use a drug efficient for both bacterial enzymes *in vivo*. Thus, good candidates could be **AAZ**, **MZA**, and **ZNS**, which have K_I_ < 400 nM against both enzymes. Of course, in the future, we will investigate other classes of CAIs for their inhibitory action against these enzymes.

## 6. Anion Inhibition Studies

**Figure 3:**
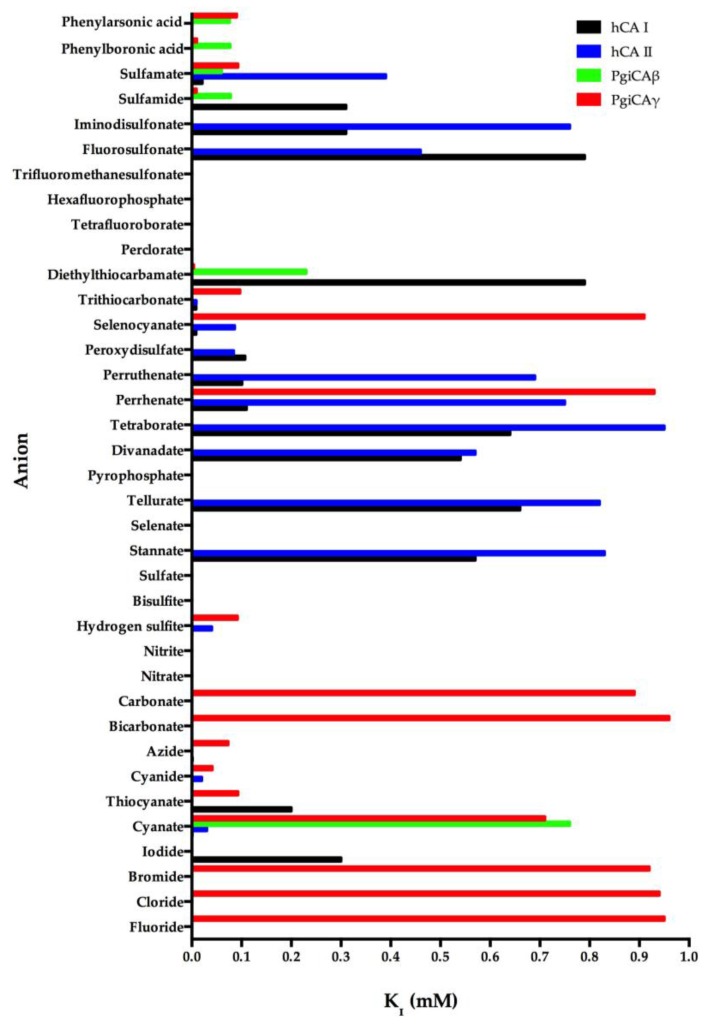
Inhibition profile of the anionic inhibitors against the α-human isoforms (hCA I and hCA II) and the two *Porphyromonas gingivalis* CAs (PgiCAβ and PgiCAγ) for the CO_2_ hydration reaction. The figure was generated using the program Prism and excludes all the anion inhibitors showing K_I_ > 1 mM. The absence of the bar means that the inhibitor exhibits K_I_ > 1 mM. The errors were in the range of 5–10% of the shown data from three different assays.

We investigated the anion inhibition profile of PgiCAβ and PgiCAγ with simple and complex anions, as well as small molecules inhibiting other CAs [19,21]. The inhibition profile of PgiCAβ is generally quite different from that of PgiCAγ, as well as the human isoform hCA II. It should be noted that perchlorate, tetrafluoroborate, azide, nitrate, hydrogensulfite, and sulfate were not inhibitors of PgiCAβ. For example, azide has an inhibition constant of 73 µM against PgiCAγ and of 1.5 mM against hCA II, but it is not at all inhibitory against PgiCAβ. Halides, cyanide, bicarbonate, nitrite, selenate, diphosphate, divanadate, tetraborate, peroxodisulfate, hexafluorophosphate, and triflate exhibit weak inhibitory activity against PgiCAβ (K_I_ of 5.4–21.4 mM) [19,21]. Interestingly, for the halogenides, fluoride and chloride show a similar behavior (K_I_ of 7.5–7.8 mM), whereas the heavier halogenides are weaker inhibitors (K_I_ of 15.9–21.4 mM). The most efficient PgiCAβ inhibitors detected so far are sulfamide, sulfamate, phenylboronic acid, and phenylarsonic acid, with K_I_ ranging between 60 and 78 µM (Figure 3). It is also interesting to note that all these compounds are also effective inhibitors of PgiCAγ, although they are far less efficient hCA II inhibitors (Figure 3). Thus, even this preliminary study was able to detect leads with good inhibitory power against the two pathogenic bacterium enzymes, in addition to good selectivity for the pathogenic over the host enzyme inhibition.

## 7. Concluding Remarks

The microbiota of the human oral mucosa consists of a myriad of bacterial species. Among them, *Porphyromonas gingivalis* is the major pathogen responsible for the development of chronic periodontitis. The *P. gingivalis* genome encodes for two CAs, one belonging to the β-CA class (PgiCAβ) and the other to the γ-CA class (PgiCAγ). These two enzymes were cloned, heterologously expressed in *Escherichia coli*, and purified to homogeneity. PgiCAβ and PgiCAγ showed significant catalytic activity for the hydration of CO_2_ to bicarbonate and protons. Several *in vitro* and *in vivo* inhibition studies with various classes of inhibitors, such as anions, sulfonamides, and sulfamates, have been reported for bacterial CAs. Efficient *in vitro* inhibitors have been discovered for many such enzymes, but only for *Neisseria spp.*, *Helicobacter pylori*, *Brucella suis*, *Streptococcus pneumoniae*, and *Mycobacterium tuberculosis* was the CA inhibition reported to lead to the inhibition of bacterial growth *in vivo*. It is thus obvious that PgiCAβ and PgiCAγ inhibition studies are needed in order to detect even stronger inhibitors, and elucidate the role that these enzymes play in the pathogenesis of *P. gingivalis* infection. In this review, the anion and sulfonamide inhibition profiles of PgiCAβ and PgiCAγ were reported. The inhibition profile of *Porphyromonas gingivalis* was very different from that of the human isozymes (h CA I and hCA II). Interestingly, the PgiCAγ enzyme was much more sensitive to sulfonamide inhibitors compared to the PgiCAβ enzyme. Moreover, PgiCAβ and PgiCAγ were antithetic towards sulfonamides. In fact, the effective inhibitors of PgiCAβ were ineffective towards PgiCAγ, and *vice versa*. PgiCAβ and PgiCAγ represent promising targets for obtaining anti-bacterials devoid of the resistance problems of the clinically used agents, but further studies are needed for the identification of potent and possibly selective inhibitors of PgiCAβ and PgiCAγ, which may lead to the development of pharmacological tools helpful in the eradication of *Porphyromonas gingivalis* that is present in chronic periodontitis.

## Figures and Tables

**Table 1:** Kinetic parameters for the CO_2_ hydration reaction catalyzed by various CAs belonging to various organisms.

Organism	Enzyme Acronym	Class	k_cat_ (s^−1^)	k_cat_/K_m_ (M^−1^ × s^−1^)
*Homo sapiens*	hCA I	α	2.0 **×** 10^5^	5.0 **×** 10^7^
	hCA II	α	1.4 **×** 10^6^	1.5 **×** 10^8^
*Flaveria bidentis*	FbCA1	β	1.2 **×** 10^5^	7.5 **×** 10^6^
*Sulfurihydrogenibium azorense*	SazCA	α	4.4 **×** 10^6^	3.5 **×** 10^8^
*Porphyromonas gingivalis*	PgiCAβ	β	2.8 **×** 10^5^	1.5 **×** 10^7^
	PgiCAγ	γ	4.1 **×** 10^5^	5.4 **×** 10^7^

Note: All data were obtained in similar conditions by a stopped-flow CO_2_ hydratase assay method. Errors in the range of 5–10% of the shown data are from three different assays.
